# The azide-bridged mixed-valent cobalt(II,III) compound [(CH_3_)_3_NH]_2_[Co^II^Co_2_^III^(N_3_)_10_]

**DOI:** 10.1107/S1600536810049421

**Published:** 2010-12-04

**Authors:** Yan-Ju Liu, Yu-Xian Li, Min Xu, Xia Wang

**Affiliations:** a Pharmacy College, Henan University of Traditional Chinese Medicine, Zhengzhou 450008, People’s Republic of China

## Abstract

The crystal structure of the title compound, poly[bis­(tri­methyl­ammonium) hexa-μ_1,1_-azido-tetra­azido­tricobalt­ate(II,III)], [(CH_3_)_3_NH]_2_[Co^II^Co^III^_2_(N_3_)_10_], consists of anionic chains [Co^II^Co^III^_2_(N_3_)_10_]^2−^ extending parallel to the *c* axis and [(CH_3_)_3_NH]^+^ counter-cations situated between the chains. In the anionic chain, one tetra­hedrally coordinated Co^II^ atom (site symmetry 2) and two octa­hedrally coordinated Co^III^ atoms are arranged alternately and are linked by μ_1,1_-azide bridges. The anionic chains and cations are connected *via* N—H⋯N hydrogen bonding into a three-dimensional structure.

## Related literature

For background to transition-metal azido-complexes templated by counter-cations of various sizes, see: Liu *et al.* (2006 ▶, 2008 ▶). For related cobalt complexes, see: Zhang *et al.* (2010 ▶).
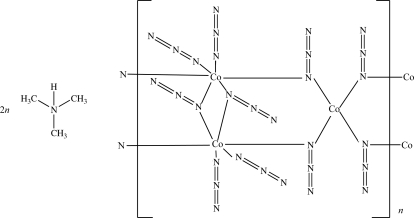


## Experimental

### 

#### Crystal data

(C_3_H_10_N)_2_[Co_3_(N_3_)_10_]
*M* *_r_* = 717.33
Monoclinic, 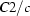

*a* = 21.7200 (6) Å
*b* = 11.3812 (4) Å
*c* = 12.1628 (4) Å
β = 115.524 (2)°
*V* = 2713.21 (15) Å^3^
*Z* = 4
Mo *K*α radiation
μ = 1.88 mm^−1^
*T* = 293 K
0.10 × 0.06 × 0.05 mm

#### Data collection

Rigaku Saturn diffractometer
Absorption correction: multi-scan (*REQAB*; Jacobson, 1998 ▶) *T*_min_ = 0.708, *T*_max_ = 0.823
22049 measured reflections
2389 independent reflections
1481 reflections with *I* > 2σ(*I*)
*R*_int_ = 0.103

#### Refinement

*R*[*F* ^2^ > 2σ(*F* ^2^)] = 0.032
*wR*(*F* ^2^) = 0.061
*S* = 0.98
2389 reflections
190 parameters
24 restraints
H-atom parameters constrained
Δρ_max_ = 0.31 e Å^−3^
Δρ_min_ = −0.35 e Å^−3^

### 

Data collection: *CrystalClear* (Rigaku/MSC, 2006 ▶); cell refinement: *CrystalClear*; data reduction: *CrystalClear*; program(s) used to solve structure: *SHELXS97* (Sheldrick, 2008 ▶); program(s) used to refine structure: *SHELXL97* (Sheldrick, 2008 ▶); molecular graphics: *SHELXTL* (Sheldrick, 2008 ▶); software used to prepare material for publication: *publCIF* (Westrip, 2010 ▶).

## Supplementary Material

Crystal structure: contains datablocks I, global. DOI: 10.1107/S1600536810049421/wm2430sup1.cif

Structure factors: contains datablocks I. DOI: 10.1107/S1600536810049421/wm2430Isup2.hkl

Additional supplementary materials:  crystallographic information; 3D view; checkCIF report

## Figures and Tables

**Table 1:** Selected bond lengths (Å)

Co1—N7	1.944 (3)
Co1—N4	1.948 (3)
Co1—N10	1.964 (3)
Co1—N1	1.979 (3)
Co1—N13^i^	2.008 (3)
Co1—N13	2.008 (3)
Co2—N1^ii^	1.968 (3)
Co2—N1	1.968 (3)
Co2—N10^i^	2.014 (3)
Co2—N10^iii^	2.014 (3)

Symmetry codes: (i) 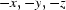; (ii) 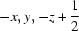; (iii) 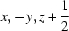.

**Table 2:** Hydrogen-bond geometry (Å, °)

*D*—H⋯*A*	*D*—H	H⋯*A*	*D*⋯*A*	*D*—H⋯*A*
N16—H16⋯N7	0.91	2.02	2.890	159

**Fig. 1.**
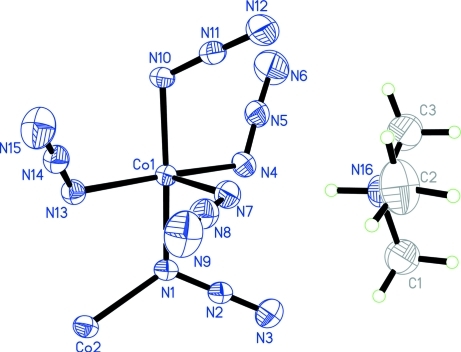
The asymmetric unit of the title structure. All non-H atoms are labelled and are shown with displacement ellipsoids at the 30% probability level. H atoms have been omitted.

**Fig. 2.**
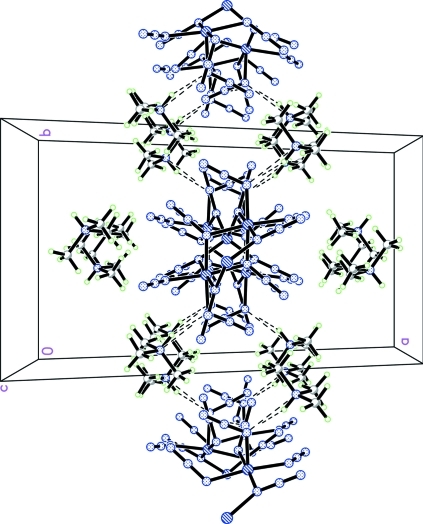
A view of the crystal packing along the c axis.

## supplementary crystallographic information

### Comment

Azido-bridged complexes have attracted a lot of attention in recent times because of their importance in diverse fields, encompassing condensed matter physics, materials chemistry, biological chemistry, *etc*. Having diverse coordination modes and being an efficient magnetic coupler, the azide anion is a versatile ligand in bridging different transition metals, generating rich and fascinating architectures ranging from discrete polynuclears to extended three-dimensional networks with interesting magnetic properties (antiferromagnetic, ferromagnetic, ferrimagnetic, canted and alternating systems). In fact, remarkable structural variations of azido-bridged complexes have been reported by using various ancillary ligands, with different number of coordination sites and steric hindrance, to control over the dimensions of complexes and bridging modes of the azide anions, thus leading to the control over their magnetic properties. However, only a few metal-azido systems devoid of ancillary ligands have been obtained by varying the size of the coutercations (Liu *et al.*, 2006, 2008). A small coutercation such as (CH_3_)_4_N^+^ produced the one-dimensional ferromagnetic complex [(CH_3_)_4_N][Cu(N_3_)_3_], in which the Cu(II) ions are connected by a triple azido-bridge, including two µ_1,3_-N_3_ and one µ_1,1_-N_3_ anions. When more bulky coutercations were employed, the mononuclear paramagnetic complex [(CH_3_CH_2_)_4_N]_2_[Cu(N_3_)_4_], the dinuclear antiferromagnetic complex [(CH_3_CH_2_CH_2_CH_2_)_4_N]_2_[Cu_2_(N_3_)_6_] and the one-dimensional ferromagnetic complex [(CH_3_CH_2_CH_2_)_4_N]_2_[Cu_3_(N_3_)_8_] were obtained separately. For magnese(II)-azido complexes, use of small coutercations like (CH_3_)_4_N^+^ and Cs^+^ produced compounds with interesting three-dimensional structures [(CH_3_)_4_N][Mn(N_3_)_3_] and Cs[Mn(N_3_)_3_], where the cations are situated in the voids the anionic Mn^II^-azido network. When using the more bulky cation (CH_3_CH_2_)_4_N^+^, the one-dimensional ferromagnetic complex [(CH_3_CH_2_)_4_N][Mn(N_3_)_3_] was obtained. Despite the results obtained above, azido-bridged complexes with mixed-valent metal ions have not been reported. In this work, we report on a mixed-valence cobalt(II,III) complex, [(CH_3_)_3_NH]_2_[Co^II^Co^III^_2_(N_3_)_10_], (I).

The structure of (I) consists of anionic chains [Co^II^Co^III^_2_(N_3_)_10_]^2-^ extending parallel to the *c*-axis. The [(CH_3_)_3_NH]^+^ countercations are situated between the chains (Fig. 1). In the [Co^II^Co^III^_2_(N_3_)_10_]^2-^ anionic chain, the Co^II^ atom (Co2, site symmetry 2) is tetrahedrally coordinated by N atoms, whereas the Co^III^ atom (Co1) is octahedrally coordinated. Co^II^ and Co^III^ atoms are linked by µ_1,1_-azido ligands and are alternatively arranged along the chain direction. The Co1—N distances range between 1.944 (3)–2.008 (3) Å, slightly longer than those expected for Co^III^. The Co2—N distances range between 1.968 (3)–2.014 (3) Å, slightly shorter than those expected for Co^II^ (Zhang *et al.*, 2010). The anionic chain and the cations are connected *via* N—H···N hydrogen bonding between the donating N—H function of the cation and non-bridging azido groups of the anion (Fig 2).

### Experimental

In a test tube a 5 ml methanol solution of 0.10*M* Co(ClO_4_)_2_^.^6H_2_O was layered carefully with 3 ml methanol and then with 10 ml methanol solution of 0.20*M* HCl, 0.20*M* NaN_3_, and 0.10*M* trimethylamine. The tube was sealed and kept undisturbed. Tiny red columnar crystals appeared overnight. Crystallization time of one week produced crystals in a yield of 25% based on the metal salt.

### Refinement

Hydrogen atoms were added geometrically and were refined using a riding model, with C—H = 0.98 Å (CH_3_) and N—H = 0.89 Å.

### Crystal data

**Table:**

(C_3_H_10_N)_2_[Co_3_(N_3_)_10_]	*F*(000) = 1444
*M**_r_* = 717.33	*D*_x_ = 1.756 Mg m^−^^3^
Monoclinic, *C*2/*c*	Mo *K*α radiation, λ = 0.71073 Å
Hall symbol: -C 2yc	Cell parameters from 14377 reflections
*a* = 21.7200 (6) Å	θ = 3.4–25.0°
*b* = 11.3812 (4) Å	µ = 1.88 mm^−^^1^
*c* = 12.1628 (4) Å	*T* = 293 K
β = 115.524 (2)°	Pillar, red
*V* = 2713.21 (15) Å^3^	0.10 × 0.06 × 0.05 mm
*Z* = 4	

### Data collection

**Table:**

Rigaku Saturn diffractometer	2389 independent reflections
Radiation source: fine-focus sealed tube	1481 reflections with *I* > 2σ(*I*)
graphite	*R*_int_ = 0.103
Detector resolution: 0.76 pixels mm^-1^	θ_max_ = 25.0°, θ_min_ = 3.6°
dtprofit.ref scans	*h* = −25→25
Absorption correction: multi-scan (*REQAB*; Jacobson, 1998)	*k* = −13→13
*T*_min_ = 0.708, *T*_max_ = 0.823	*l* = −14→14
22049 measured reflections	

### Refinement

**Table:**

Refinement on *F*^2^	Secondary atom site location: difference Fourier map
Least-squares matrix: full	Hydrogen site location: inferred from neighbouring sites
*R*[*F*^2^ > 2σ(*F*^2^)] = 0.032	H-atom parameters constrained
*wR*(*F*^2^) = 0.061	*w* = 1/[σ^2^(*F*_o_^2^) + (0.0204*P*)^2^] where *P* = (*F*_o_^2^ + 2*F*_c_^2^)/3
*S* = 0.98	(Δ/σ)_max_ = 0.001
2389 reflections	Δρ_max_ = 0.31 e Å^−^^3^
190 parameters	Δρ_min_ = −0.35 e Å^−^^3^
24 restraints	Extinction correction: *SHELXL97* (Sheldrick, 2008), Fc^*^=kFc[1+0.001xFc^2^λ^3^/sin(2θ)]^-1/4^
Primary atom site location: structure-invariant direct methods	Extinction coefficient: 0.00084 (18)

### Special details

**Table:**

Geometry. All e.s.d.'s (except the e.s.d. in the dihedral angle between two l.s. planes) are estimated using the full covariance matrix. The cell e.s.d.'s are taken into account individually in the estimation of e.s.d.'s in distances, angles and torsion angles; correlations between e.s.d.'s in cell parameters are only used when they are defined by crystal symmetry. An approximate (isotropic) treatment of cell e.s.d.'s is used for estimating e.s.d.'s involving l.s. planes.
Refinement. Refinement of *F*^2^ against ALL reflections. The weighted *R*-factor *wR* and goodness of fit *S* are based on *F*^2^, conventional *R*-factors *R* are based on *F*, with *F* set to zero for negative *F*^2^. The threshold expression of *F*^2^ > σ(*F*^2^) is used only for calculating *R*-factors(gt) *etc*. and is not relevant to the choice of reflections for refinement. *R*-factors based on *F*^2^ are statistically about twice as large as those based on *F*, and *R*- factors based on ALL data will be even larger.

### Fractional atomic coordinates and isotropic or equivalent isotropic displacement parameters (Å^2^)

**Table:**

	*x*	*y*	*z*	*U*_iso_*/*U*_eq_	
Co1	0.04965 (2)	0.10300 (4)	0.05706 (4)	0.03529 (17)	
Co2	0.0000	−0.04737 (5)	0.2500	0.0402 (2)	
N1	0.06948 (14)	0.0409 (2)	0.2209 (3)	0.0428 (6)	
N2	0.12002 (17)	0.0827 (3)	0.3042 (3)	0.0448 (6)	
N3	0.16669 (18)	0.1210 (3)	0.3810 (3)	0.0740 (10)	
N4	0.14720 (14)	0.1256 (2)	0.1097 (3)	0.0523 (7)	
N5	0.16894 (15)	0.1348 (3)	0.0363 (3)	0.0540 (7)	
N6	0.1925 (2)	0.1442 (4)	−0.0308 (4)	0.1069 (15)	
N7	0.04578 (16)	0.2621 (2)	0.1120 (3)	0.0489 (8)	
N8	−0.00656 (18)	0.3009 (3)	0.1097 (3)	0.0504 (8)	
N9	−0.05415 (18)	0.3426 (3)	0.1100 (3)	0.0806 (12)	
N10	0.02748 (13)	0.1593 (3)	−0.1084 (2)	0.0434 (6)	
N11	0.04393 (15)	0.2609 (3)	−0.1156 (2)	0.0492 (6)	
N12	0.0599 (2)	0.3555 (3)	−0.1195 (3)	0.0930 (13)	
N13	−0.04827 (13)	0.0595 (2)	0.0083 (2)	0.0384 (6)	
N14	−0.09288 (16)	0.1253 (3)	−0.0627 (3)	0.0425 (6)	
N15	−0.13450 (17)	0.1858 (3)	−0.1255 (3)	0.0715 (10)	
N16	0.16193 (14)	0.4179 (3)	0.2144 (3)	0.0539 (8)	
H16	0.1336	0.3554	0.1828	0.065*	
C1	0.2022 (2)	0.3949 (5)	0.3459 (4)	0.1044 (17)	
H1A	0.2292	0.4628	0.3840	0.157*	
H1B	0.2316	0.3287	0.3565	0.157*	
H1C	0.1719	0.3783	0.3826	0.157*	
C2	0.1190 (3)	0.5217 (4)	0.1950 (5)	0.1125 (19)	
H2A	0.0852	0.5067	0.2242	0.169*	
H2B	0.0970	0.5396	0.1096	0.169*	
H2C	0.1468	0.5871	0.2384	0.169*	
C3	0.2062 (2)	0.4251 (5)	0.1509 (4)	0.1121 (19)	
H3A	0.1787	0.4379	0.0655	0.168*	
H3B	0.2311	0.3529	0.1621	0.168*	
H3C	0.2377	0.4891	0.1837	0.168*	

### Atomic displacement parameters (Å^2^)

**Table:**

	*U*^11^	*U*^22^	*U*^33^	*U*^12^	*U*^13^	*U*^23^
Co1	0.0382 (3)	0.0364 (3)	0.0331 (3)	−0.0041 (2)	0.0171 (2)	−0.0013 (2)
Co2	0.0487 (4)	0.0370 (4)	0.0412 (5)	0.000	0.0253 (4)	0.000
N1	0.0456 (15)	0.0502 (15)	0.0338 (16)	−0.0052 (12)	0.0181 (13)	0.0022 (12)
N2	0.0475 (15)	0.0518 (15)	0.0353 (16)	−0.0042 (13)	0.0180 (13)	0.0033 (13)
N3	0.065 (2)	0.095 (3)	0.052 (2)	−0.019 (2)	0.015 (2)	−0.006 (2)
N4	0.0449 (15)	0.0647 (16)	0.0528 (17)	−0.0104 (12)	0.0264 (12)	−0.0027 (13)
N5	0.0453 (15)	0.0637 (16)	0.0561 (18)	−0.0079 (12)	0.0249 (13)	−0.0055 (13)
N6	0.097 (3)	0.148 (4)	0.112 (4)	−0.027 (3)	0.079 (3)	−0.026 (3)
N7	0.061 (2)	0.0407 (19)	0.051 (2)	−0.0085 (16)	0.0293 (18)	−0.0088 (15)
N8	0.062 (2)	0.040 (2)	0.050 (2)	−0.0019 (17)	0.025 (2)	−0.0050 (15)
N9	0.071 (3)	0.068 (3)	0.103 (3)	0.009 (2)	0.037 (3)	−0.020 (2)
N10	0.0570 (15)	0.0435 (16)	0.0329 (14)	−0.0095 (13)	0.0222 (12)	0.0004 (13)
N11	0.0626 (15)	0.0478 (16)	0.0348 (15)	−0.0086 (14)	0.0186 (12)	0.0007 (13)
N12	0.150 (4)	0.054 (2)	0.064 (3)	−0.036 (2)	0.036 (3)	0.004 (2)
N13	0.0378 (15)	0.0388 (15)	0.0414 (17)	−0.0002 (10)	0.0197 (13)	−0.0025 (11)
N14	0.0408 (15)	0.0412 (16)	0.0447 (17)	−0.0017 (11)	0.0176 (13)	−0.0036 (11)
N15	0.058 (2)	0.058 (2)	0.081 (3)	0.0085 (19)	0.014 (2)	0.005 (2)
N16	0.0501 (19)	0.050 (2)	0.061 (2)	−0.0163 (15)	0.0234 (19)	−0.0118 (16)
C1	0.083 (3)	0.143 (5)	0.067 (4)	−0.039 (3)	0.013 (3)	0.007 (3)
C2	0.115 (4)	0.052 (3)	0.165 (6)	0.015 (3)	0.055 (4)	−0.001 (3)
C3	0.093 (4)	0.173 (5)	0.099 (4)	−0.055 (4)	0.069 (3)	−0.045 (4)

### Geometric parameters (Å, °)

**Table:**

Co1—N7	1.944 (3)	N11—N12	1.139 (4)
Co1—N4	1.948 (3)	N13—N14	1.234 (4)
Co1—N10	1.964 (3)	N13—Co1^i^	2.008 (3)
Co1—N1	1.979 (3)	N14—N15	1.131 (4)
Co1—N13^i^	2.008 (3)	N16—C2	1.460 (5)
Co1—N13	2.008 (3)	N16—C3	1.473 (4)
Co2—N1^ii^	1.968 (3)	N16—C1	1.478 (5)
Co2—N1	1.968 (3)	N16—H16	0.9100
Co2—N10^i^	2.014 (3)	C1—H1A	0.9600
Co2—N10^iii^	2.014 (3)	C1—H1B	0.9600
N1—N2	1.224 (4)	C1—H1C	0.9600
N2—N3	1.129 (4)	C2—H2A	0.9600
N4—N5	1.181 (4)	C2—H2B	0.9600
N5—N6	1.140 (4)	C2—H2C	0.9600
N7—N8	1.209 (4)	C3—H3A	0.9600
N8—N9	1.139 (4)	C3—H3B	0.9600
N10—N11	1.225 (4)	C3—H3C	0.9600
N10—Co2^i^	2.014 (3)		
			
N7—Co1—N4	88.05 (12)	Co1—N10—Co2^i^	121.43 (14)
N7—Co1—N10	91.18 (12)	N12—N11—N10	178.5 (4)
N4—Co1—N10	92.62 (12)	N14—N13—Co1^i^	114.1 (2)
N7—Co1—N1	90.55 (12)	N14—N13—Co1	118.0 (2)
N4—Co1—N1	88.97 (12)	Co1^i^—N13—Co1	100.22 (11)
N10—Co1—N1	177.69 (12)	N15—N14—N13	178.2 (4)
N7—Co1—N13^i^	176.56 (13)	C2—N16—C3	112.7 (4)
N4—Co1—N13^i^	94.61 (11)	C2—N16—C1	110.9 (4)
N10—Co1—N13^i^	86.53 (11)	C3—N16—C1	111.2 (3)
N1—Co1—N13^i^	91.67 (11)	C2—N16—H16	107.3
N7—Co1—N13	97.71 (11)	C3—N16—H16	107.3
N4—Co1—N13	173.16 (12)	C1—N16—H16	107.3
N10—Co1—N13	90.96 (11)	N16—C1—H1A	109.5
N1—Co1—N13	87.29 (11)	N16—C1—H1B	109.5
N13^i^—Co1—N13	79.78 (11)	H1A—C1—H1B	109.5
N1^ii^—Co2—N1	118.61 (16)	N16—C1—H1C	109.5
N1^ii^—Co2—N10^i^	120.58 (11)	H1A—C1—H1C	109.5
N1—Co2—N10^i^	97.86 (11)	H1B—C1—H1C	109.5
N1^ii^—Co2—N10^iii^	97.86 (11)	N16—C2—H2A	109.5
N1—Co2—N10^iii^	120.58 (11)	N16—C2—H2B	109.5
N10^i^—Co2—N10^iii^	101.58 (16)	H2A—C2—H2B	109.5
N2—N1—Co2	122.3 (2)	N16—C2—H2C	109.5
N2—N1—Co1	115.0 (2)	H2A—C2—H2C	109.5
Co2—N1—Co1	120.82 (15)	H2B—C2—H2C	109.5
N3—N2—N1	179.9 (5)	N16—C3—H3A	109.5
N5—N4—Co1	119.7 (3)	N16—C3—H3B	109.5
N6—N5—N4	177.2 (4)	H3A—C3—H3B	109.5
N8—N7—Co1	120.8 (2)	N16—C3—H3C	109.5
N9—N8—N7	176.5 (4)	H3A—C3—H3C	109.5
N11—N10—Co1	115.5 (2)	H3B—C3—H3C	109.5
N11—N10—Co2^i^	121.6 (2)		

Symmetry codes: (i) −*x*, −*y*, −*z*; (ii) −*x*, *y*, −*z*+1/2; (iii) *x*, −*y*, *z*+1/2.

### Hydrogen-bond geometry (Å, °)

**Table:**

*D*—H···*A*	*D*—H	H···*A*	*D*···*A*	*D*—H···*A*
N16—H16···N7	0.91	2.02	2.890	159.
